# A3 CELIAC DISEASE AUTOIMMUNITY INCREASING AMONG VARIOUS PEDIATRIC SUBGROUPS IN ALBERTA, CANADA

**DOI:** 10.1093/jcag/gwae059.003

**Published:** 2025-02-10

**Authors:** J A King, S Coward, J Godley, A Metcalfe, P Ronksley, G G Kaplan, T Williamson

**Affiliations:** Community Health Sciences, University of Calgary, Calgary, AB, Canada; Community Health Sciences, University of Calgary, Calgary, AB, Canada; Community Health Sciences, University of Calgary, Calgary, AB, Canada; Community Health Sciences, University of Calgary, Calgary, AB, Canada; Community Health Sciences, University of Calgary, Calgary, AB, Canada; Community Health Sciences, University of Calgary, Calgary, AB, Canada; Community Health Sciences, University of Calgary, Calgary, AB, Canada

## Abstract

**Background:**

Celiac disease (CeD) autoimmunity, defined as elevated tissue transglutaminase (TTG) levels with or without biopsy confirmation, is rising among children in Canada; however, further investigation is needed to understand if this varies across pediatric subpopulations.

**Aims:**

To evaluate incidence and trends in CeD autoimmunity among different pediatric subgroups.

**Methods:**

Using population-based administrative data, we identified individuals under 15 years old during their first positive TTG test in Alberta from April 2015 to March 2024. Based on the year of test, we determined socioeconomic status by matching patient postal code to census level dissemination area (which translate to material and social deprivation quintiles) and geographic residence. Incidence per 100,000 person-years (PY) and 95% confidence intervals (CIs) were calculated for subgroups (sex, age, rurality, material and social deprivation) within time periods (pre-pandemic [2015–2019], pandemic [2020–2022], and post-pandemic [2023–2024]). Incidence rate ratios (IRRs) were estimated to compare pre-pandemic and post-pandemic rates.

**Results:**

A total of 5,141 children with incident CeD autoimmunity were identified. The highest incidence was observed post-pandemic (92 per 100,000 PY), particularly among those aged 5–9 years (135 per 100,000 PY), those living in the least materially deprived areas (117 per 100,000 PY), and females (113 per 100,000 PY). All subgroups saw significantly higher rates in the post-pandemic period relative to the pre-pandemic period, except for those most materially deprived (IRR = 1.1 [95% CI: 0.9, 1.4]). A larger increase in rates was seen in those most materially privileged (IRR = 1.7 [95% CI: 1.5, 2.0]). A change in incidence was also more pronounced among children in rural (IRR = 1.7 [95% CI: 1.5, 1.9]) compared to those in urban areas (IRR = 1.3 [95% CI: 1.2, 1.4]).

**Conclusions:**

Although rates of CeD autoimmunity vary between pediatric subpopulations, most groups are experiencing significant increases over time. This includes populations historically experiencing lower rates (e.g., those in rural areas), indicating a mounting burden of CeD for the future.

Incidence rates for CeD autoimmunity across subgroups and time

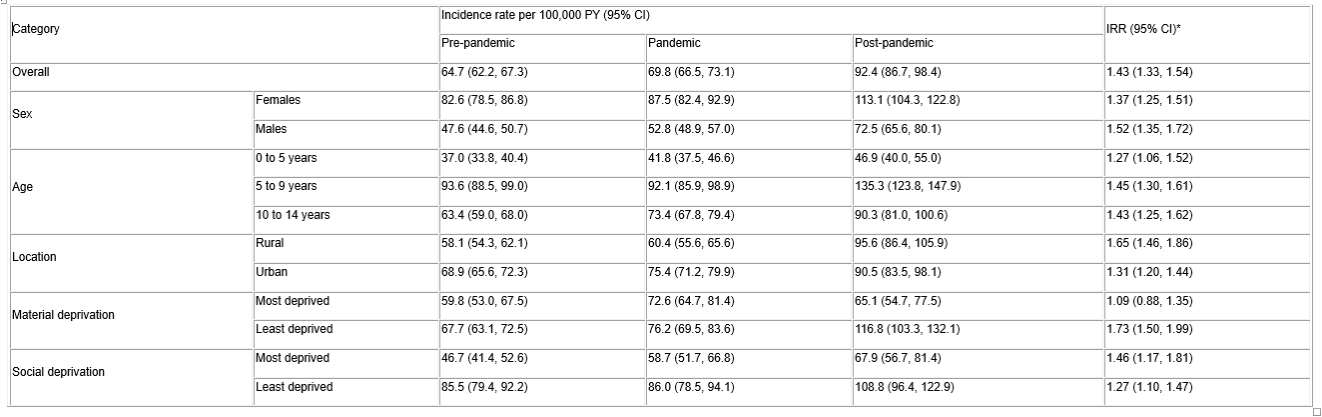

*comparing post-pandemic to pre-pandemic

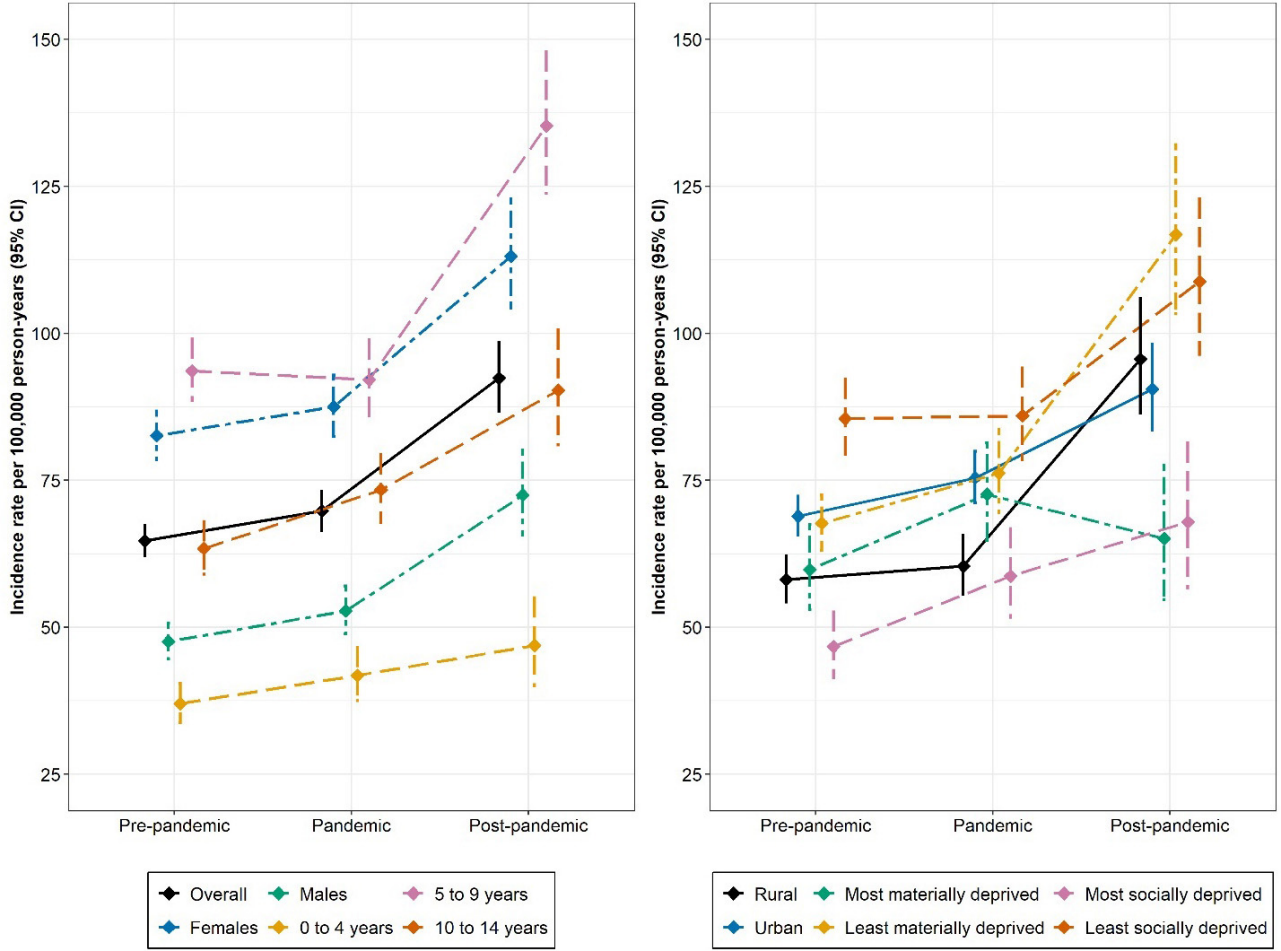

**Funding Agencies:**

CIHRTRIANGLE (Training a new generation of researchers in gastroenterology and liver)

